# Tissue engineering the human auricle by auricular chondrocyte-mesenchymal stem cell co-implantation

**DOI:** 10.1371/journal.pone.0202356

**Published:** 2018-10-24

**Authors:** Benjamin P. Cohen, Jaime L. Bernstein, Kerry A. Morrison, Jason A. Spector, Lawrence J. Bonassar

**Affiliations:** 1 Nancy E. and Peter C. Meinig School of Biomedical Engineering, Cornell University, Ithaca, New York, United States of America; 2 Division of Plastic Surgery, Weill Cornell Medical College, New York, New York, United States of America; 3 Sibley School of Mechanical and Aerospace Engineering, Cornell University, Ithaca, New York, United States of America; University of Umeå, SWEDEN

## Abstract

Children suffering from microtia have few options for auricular reconstruction. Tissue engineering approaches attempt to replicate the complex anatomy and structure of the ear with autologous cartilage but have been limited by access to clinically accessible cell sources. Here we present a full-scale, patient-based human ear generated by implantation of human auricular chondrocytes and human mesenchymal stem cells in a 1:1 ratio. Additional disc construct surrogates were generated with 1:0, 1:1, and 0:1 combinations of auricular chondrocytes and mesenchymal stem cells. After 3 months *in vivo*, monocellular auricular chondrocyte discs and 1:1 disc and ear constructs displayed bundled collagen fibers in a perichondrial layer, rich proteoglycan deposition, and elastin fiber network formation similar to native human auricular cartilage, with the protein composition and mechanical stiffness of native tissue. Full ear constructs with a 1:1 cell combination maintained gross ear structure and developed a cartilaginous appearance following implantation. These studies demonstrate the successful engineering of a patient-specific human auricle using exclusively human cell sources without extensive *in vitro* tissue culture prior to implantation, a critical step towards the clinical application of tissue engineering for auricular reconstruction.

## Introduction

For over two decades, tissue engineering the human auricle, or external ear, has been pursued as an alternative to existing methods of auricular reconstruction [1]. The current gold standard treatment for patients with significant deformation or damage of the auricle is autologous reconstruction using costal cartilage. This is a complex surgical technique employed by relatively few surgeons due to morbidity at the rib cartilage donor site and challenges in producing auricles with acceptable aesthetic results [2–4]. Although there are reports of successful reconstruction using prosthetic scaffolds, widespread adoption of this approach has been limited by poor biocompatibility and potential for extrusion [3]. These challenges have spurred interest in tissue engineering full-scale human auricles. Seeding auricular chondrocytes (AuCs) onto natural and synthetic scaffolds has generated tissue of various dimensions *in vivo* matching the structural [3,5–8], biochemical [3,5–7], and mechanical [5,6] properties of native auricular cartilage. Tissue engineered auricles can also exactly replicate the patient-specific auricular anatomy by combining non-invasive imaging modalities with computer-assisted design/computer-aided manufacturing (CAD/CAM) technology [5,6,9], offering optimal aesthetic results.

Like the autologous reconstruction currently in practice, tissue engineering utilizes autologous cells from the patient to form the desired tissue, eliminating the risk of immune rejection. Currently, autologous articular chondrocytes are isolated, expanded, and re-implanted to repair focal defects of the articular cartilage, requiring the generation of less than 1 mL of tissue [10]. Auricular cartilage can be engineered in a similar manner, however, a full-sized pediatric ear requires over 200 million cells and is ~10 mL in volume [11]. Monolayer expansion of isolated chondrocytes can result in dedifferentiation, limiting the capacity to generate robust cartilage [2,12], and potentially requires extensive 3D construct culture prior to implantation [9]. Alternatively, mesenchymal stem cells (MSCs) are multipotent cells capable of differentiation into chondrocytes, and can be readily obtained from bone marrow and expanded [13–15]. One method of using MSCs for cartilage generation is through co-culture or co-implantation of the MSCs with the desired cell phenotype [16]. Co-culture of MSCs with various chondrocyte phenotypes generated cartilage tissue while reducing the chondrocyte requirement [17–20]. However, little is known about the behavior of AuCs in combination with MSCs. The co-implantation of AuCs with MSCs [21–23] or adipose-derived stem cells [24] has generated cartilage *in vivo*, yet the impact of these studies is limited due to the use of non-human cells [21–23,25], a lack of markers specific to elastic cartilage [21,22], or the absence of mechanical evaluation [22,24].

Here we use a combination of patient-derived AuCs and MSCs to generate human ear cartilage that matches the anatomic features, structure, composition, and mechanics of native auricular cartilage. We demonstrate that suitable amounts of human auricular cartilage can be acquired from standard otoplasty procedures, and that a sufficient number of AuCs can be isolated and expanded from this tissue to form a full-sized ear construct. Additionally, we assessed whether the expanded AuCs could generate auricular cartilage *in vivo*, either alone or in combination with human MSCs, and if MSCs alone can differentiate and produce cartilage in the subcutaneous environment. Finally, we formed human-shaped ear constructs containing a 1:1 combination of AuCs and MSCs and investigated the shape retention and development of auricular cartilage structure following immediate implantation, without extensive *in vitro* culture. We report a robust and rapid process to generate anatomically shaped auricles using cells of exclusively human sources, demonstrating a clinically relevant tissue engineering alternative to autologous or alloplastic auricular reconstruction.

## Results

### Generation of full-sized human auricles from clinical cartilage remnants

**Fig 1:**
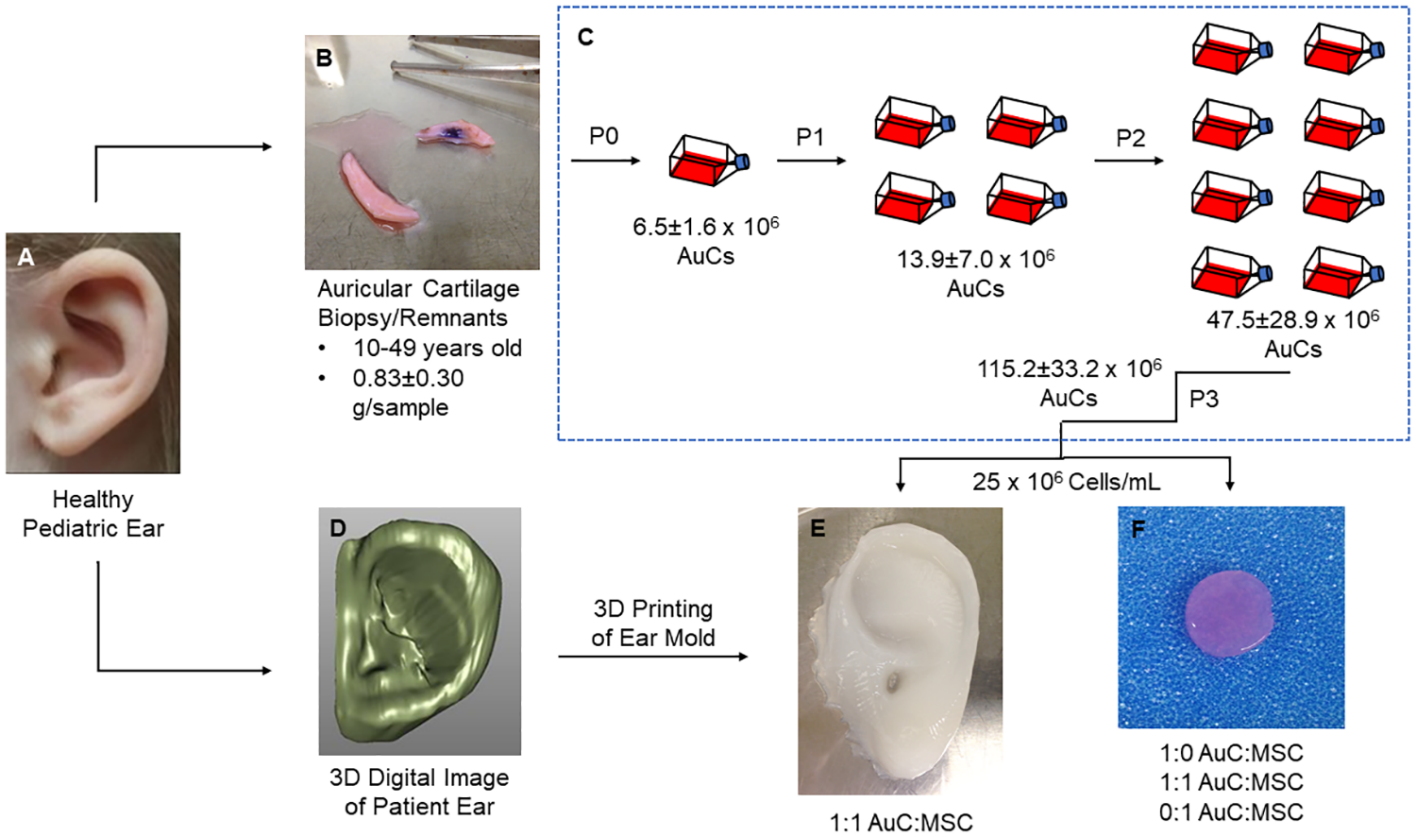
Human auricular cartilage engineering process. (**A**) High-resolution images of a pediatric ear were rapidly scanned, while human auricular cartilage was obtained from healthy donors. (**B**) The remnants of otoplasty procedures were cleaned of perichondrium and damaged tissue before digestion in collagenase. (**C**) Human AuCs were plated and expanded through passage 3. All data shown ± SD. (**D**) Images of the patient ear were converted to a continuous digital surface and edited to remove noise and enhance definition of external features. (**E**) Human AuCs were combined in a 1:1 ratio with human MSCs, encapsulated in type I collagen, and injected into patient-specific molds which were 3D printed based on the digital images. (**F**) Human AuCs and human MSCs were combined in ratios of 1:0, 1:1, and 0:1 AuC:MSC, encapsulated in type I collagen, and polymerized to form disc constructs 8 mm in diameter and 2 mm in thickness.

To demonstrate the capacity of patient-derived cells to generate ear cartilage, we combined our existing methods for auricular cartilage engineering with relevant cells of human origin. Cartilage samples for AuC isolation were derived from discarded otoplasty specimens (Fig 1A and 1B). Ear cartilage remnants were obtained from 8 healthy patients (S1 Table) courtesy of the private practices of Drs. Charles Thorne and John Sherman with informed consent, exempt from IRB approval. Cells were isolated within 24 hours of surgery.

Human AuCs were expanded through third passage (P3) before being encapsulated within engineered constructs. An average of 0.83±0.30 g (n = 8) of human auricular cartilage tissue was procured from otoplasty remnants (Fig 1B), from which an average of 8.3±2.3 million AuCs/g of tissue were isolated. By P3, the number of AuCs was 17.8 times the initial number of cells isolated (*P* < 0.05), for an average of over 115 million cells (Fig 1C). In parallel, we purchased and cultured commercially available human MSCs (RoosterBio Inc., Frederick, MD).

AuCs and MSCs were encapsulated into engineered constructs for two related studies. First, expanded AuCs were combined with MSCs to form disc constructs to compare monocellular to combined cellular implantation and to investigate progressive development over time. Discs were formed containing ratios of 1:0, 1:1, and 0:1 AuC:MSC to act as ear cartilage surrogates (Fig 1F). In the second study, full-scale, pediatric ear constructs were formed by combining AuCs and MSCs in a 1:1 ratio. Expansion of human AuCs resulted in greater than 100 million cells from a single patient’s donor tissue through P3, enough cells to generate a human ear construct when combined with human MSCs at a 1:1 cell ratio. To replicate the patient-specific morphology, we used established methods whereby scans of a normal pediatric ear were obtained and processed into a 3D digital surface (Fig 1D) which provided the negative space for an ear mold [11]. The molds were designed and 3D printed in several pieces to allow simple removal of generated constructs, as previously described [11]. All constructs were formed from high-density (10 mg/mL) type I collagen, which was chosen for its strong mechanical properties, limited contraction, and high cell viability and remodeling capacity [26,27]. For the 1:1 ears, AuCs were combined with MSCs then encapsulated in the collagen hydrogel, before being injected into the pediatric ear molds to form constructs (Fig 1E). Constructs were implanted subcutaneously into athymic nude mice (discs) or rats (ears) to provide an environment for *in vivo* maturation [1]. Animal care and experimental procedures were conducted under the guidelines of the Weill Cornell Medical College Institutional Animal Care and Use Committee.

**Fig 2:**
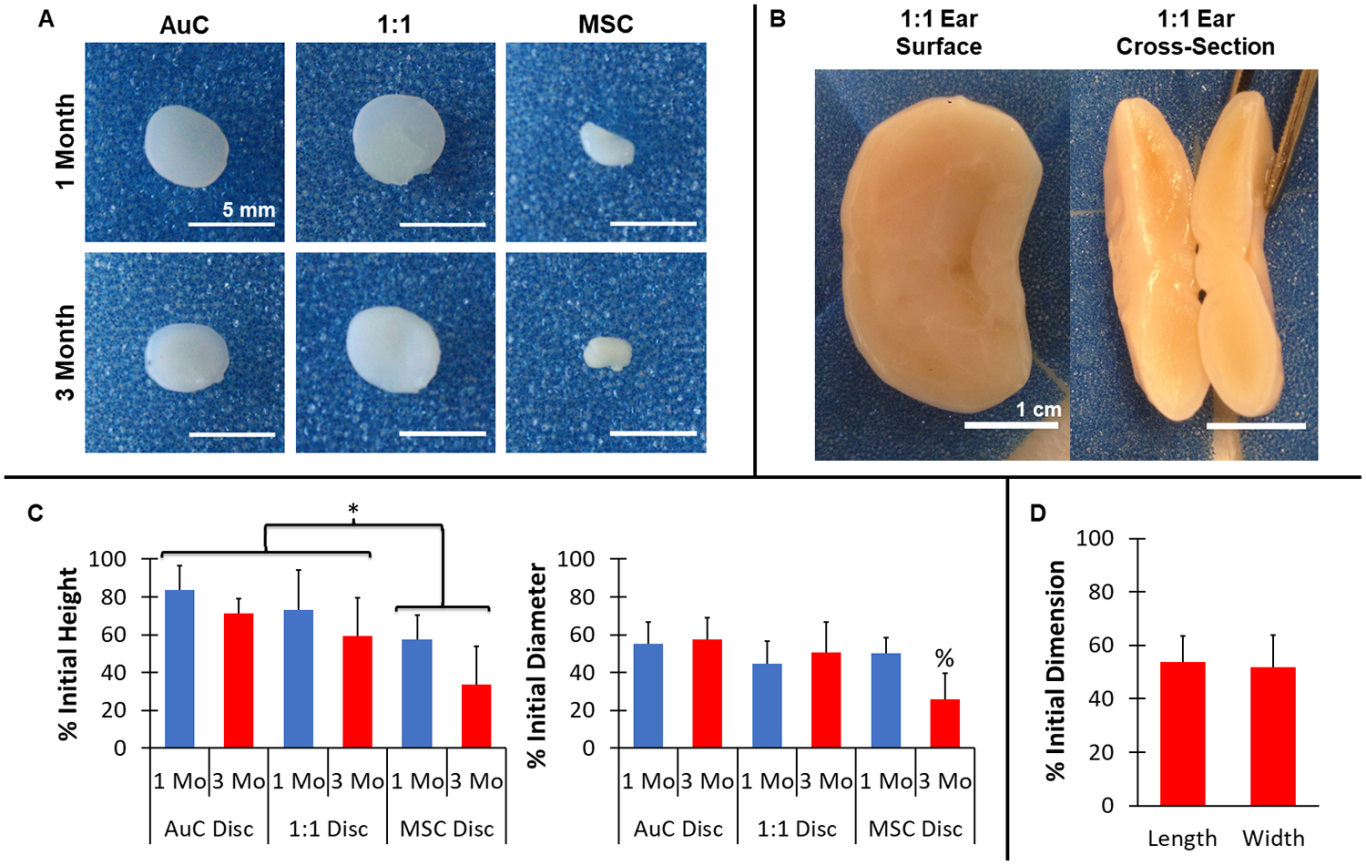
Gross examination of engineered cartilage. (**A**) *Ex vivo* gross analysis of engineered disc constructs. AuC and 1:1 AuC:MSC co-implanted constructs maintained cylindrical geometry and developed white, cartilage-like appearance after 1 and 3 months, while MSC discs contracted significantly. Scale bar = 5 mm. (**B**) *Ex vivo* gross analysis of full-scale human ear constructs formed with 1:1 AuCs to MSCs cell combination. Ear constructs maintained anatomic fidelity following 3 months *in vivo* and displayed no evidence of necrosis through the full thickness of the tissue. Scale bar = 1 cm. (**C**) AuC and 1:1 discs were significantly larger in height compared to MSC discs, and height decreased significantly in time for all groups. At 3 months, the diameter of the MSC discs was significantly less than AuC and 1:1 discs. * indicates significant difference between cell groups, % indicates significant difference between cell groups at 3 months, n = 8–9, *P* < 0.05 (**D**) 1:1 AuC:MSC full-scale ear constructs contracted in both length and width after 3 months *in vivo*. n = 7. Data in (C) and (D) displayed as mean + SD.

Following implantation, constructs containing AuC monoculture and 1:1 combination of AuCs and MSCs maintained initial morphology and developed a cartilage-like appearance and texture, while discs containing MSCs alone demonstrated poor tissue development and did not maintain their shape (Fig 2A and 2B). At both 1 and 3 months, AuC and 1:1 discs retained the original cylindrical geometry, and featured shiny, off-white color similar to auricular cartilage. Qualitatively, AuC and 1:1 discs demonstrated an elastic flexibility when handled. In contrast, the MSC discs contracted, with some approaching a spherical morphology, and displayed a rough exterior with little flexibility, more closely resembling fibrotic tissue rather than cartilage. Similar to the 1:1 disc surrogates, full-scale 1:1 ear constructs generated healthy cartilage tissue and maintained overall ear morphology after 3 months *in vivo* (Fig 2B). The 1:1 ears featured a shiny, stiffened surface, and demonstrated elastic flexibility when handled and bent (S1 Movie). In addition, cross-sections of the ear constructs displayed formation of thick, robust cartilage-like tissue throughout the implant, with thicknesses as great as 1 cm (Fig 2B).

While all constructs contracted from initial dimensions, explanted AuC and 1:1 discs featured significantly better maintenance of construct height and diameter compared to MSC discs. All discs were generated with a height of 2 mm and diameter of 8 mm. Following explantation, AuC and 1:1 discs showed superior retention in disc height and diameter compared to the discs containing MSCs alone (Fig 2C, *P* < 0.05). After 3 months, AuC discs were 71% and 57% of the initial height and diameter, respectively, while 1:1 discs were 59% and 52%. MSC discs displayed extreme contraction to 34% and 26% of original disc height and diameter after 3 months, significantly more than the AuC or 1:1 groups (*P* < 0.05). Additionally, all disc heights were lower at 3 months compared to 1 month (*P* < 0.05). Similarly, the 1:1 ear constructs experienced contraction during *in vivo* maturation. Ear construct length, measured along the lobule-helix axis, was 4.6 mm prior to implantation. The width of the ear, measured along the largest dimension perpendicular to the height axis [11], was initially 2.9 mm. Following 3 months *in vivo*, the length and width of the full-scale ear constructs decreased 54% and 52%, respectively (Fig 2D), which was more severe than the contraction observed in ears previously formed with bovine cells [11]. Despite these changes in dimension, the overall auricular shape of the construct was retained, in particular important features such as the helical rim, the anti-helix, and the lobule.

### Auricular cartilage microstructure in full-size human auricles

A major challenge of engineering a large amount of cartilage is replicating the biochemical content and structure. Following the evaluation of the construct macrostructure, we next investigated whether the engineered tissues also displayed the micro-scale structure of auricular cartilage. Auricular cartilage extracellular matrix (ECM) is composed primarily of collagen, proteoglycans, and specific to elastic cartilage, a fibrous elastin network.

**Fig 3:**
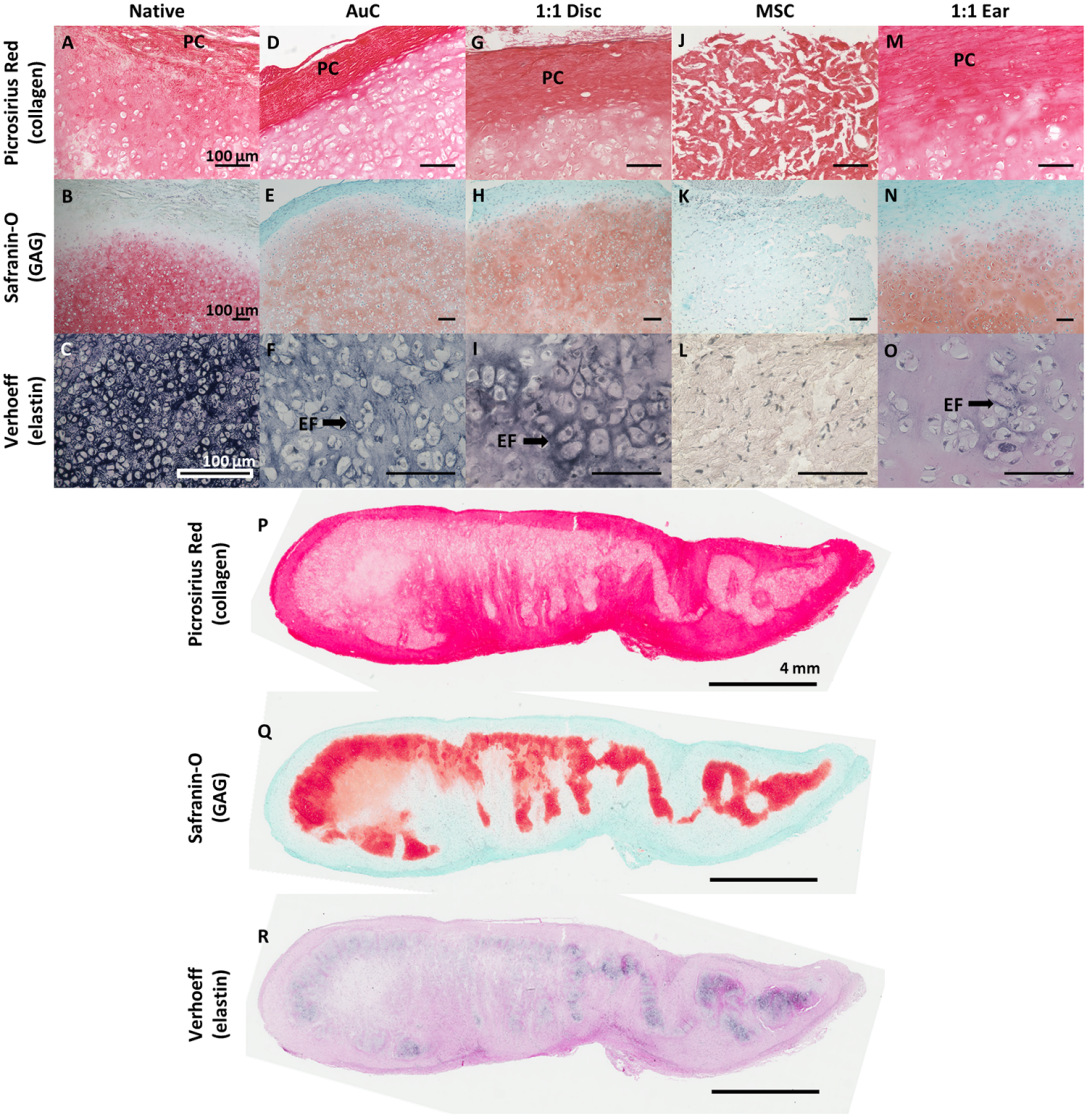
Recapitulation of auricular cartilage microstructure. Histological staining of native human auricular cartilage (**A-C**), disc constructs (**D-L**), and ear constructs (**M-R**) following 3 months *in vivo*. Picrosirius Red staining (**A, D, G, J, M, P**) displayed the formation of a perichondrium (PC) composed of collagen fibers at the perimeter of AuC and 1:1 disc and 1:1 ear constructs similar to native cartilage, while MSC discs were composed of fibrous collagen throughout. Safranin O staining with Fast Green counterstain (**B, E, H, K, N, Q**) displayed proteoglycan deposition and cell lacunae formation in AuC and 1:1 disc and 1:1 ear constructs similar to native cartilage, with no proteoglycan deposition in MSC discs. Verhoeff’s stain (**C, F, I, L, O, R**) displayed the formation of a maturing elastin fiber (EF) network in AuC and 1:1 discs similar to native cartilage, with less mature fibers appearing in 1:1 ear constructs. No elastin was observed in MSC discs. Scale bar = 100 μm for (**A-O**) and 4 mm for (**P-R**).

Engineered AuC discs, 1:1 discs, and 1:1 ear constructs displayed auricular cartilage microstructure similar to native tissue after 3 months *in vivo*. Native human auricular cartilage featured a collagen-rich perichondrial surface layer (Fig 3A), a central proteoglycan-rich tissue containing cellular lacunae (Fig 3B), and a dense network of elastin fibers surrounding the cells and spreading throughout the tissue (Fig 3C). Disc constructs containing AuCs or the 1:1 combination featured a similar structure, including the formation of the perichondrial layer (Fig 3D and 3G), deposition of proteoglycans and formation of cell lacunae (Fig 3E and 3H), and development of an elastin fiber network (Fig 3F and 3I). By comparison, MSC discs completely lacked auricular cartilage formation. MSC discs were devoid of proteoglycans (Fig 3K) or elastin (Fig 3L), with only fibrous collagen remaining after 3 months (Fig 3J), indicating limited remodeling of the initial collagen matrix. AuC and 1:1 discs at 1 month displayed the formation of perichondrial layers and proteoglycan deposition, although elastin fiber formation was limited (S1 Fig). MSC discs at 1 month featured no evidence of cartilage development.

Full-scale ear constructs containing a 1:1 combination of AuCs and MSCs also developed microstructural components closely resembling native auricular cartilage after 3 months, with evidence of healthy development throughout the nearly 1 cm thick tissue. Ear constructs featured a collagen-rich perichondrial layer (Fig 3M) and proteoglycan staining with cell lacunae (Fig 3N), similar in structure to native tissue, AuC discs, and 1:1 disc constructs. Following 3 months *in vivo*, the 1:1 ear constructs demonstrated staining for elastin fiber formation, however this was not as dense as native cartilage or the smaller disc constructs (Fig 3O). As the full ear constructs were much larger than the discs, we analyzed the full cross-section of the ears. The ear constructs demonstrated consistent formation of the fibrous perichondrial layer around the whole ear (Fig 3P). Staining for proteoglycans (Fig 3Q) and elastin (Fig 3R) was present heterogeneously throughout the tissue.

### Composition and mechanical properties of full-scale ears match those of native auricular cartilage

The ear undergoes mechanical loading in the form of tension, compression, and bending under normal physiological conditions. As such, it is critical to demonstrate the mechanical properties and stability of engineered ear cartilage, which are directly related to the ECM composition of the tissue. Engineered constructs were analyzed for DNA content, representing cell concentration, [28] and the ECM components proteoglycans, measured by sulfated glycosaminoglycans (GAGs) [29], and collagen and elastin, measured by hydroxyproline [30,31]. DNA content was higher for all engineered tissues compared to native cartilage, although this difference was not significant (S2 Fig, *P* = 0.39). The water composition was significantly different between constructs, with MSC discs significantly less hydrated after 3 months when compared to AuC or 1:1 discs or 1:1 ears (S2 Fig, *P* < 0.05). We also measured the equilibrium modulus and hydraulic permeability of the tissue in confined compression [23,32].

**Fig 4:**
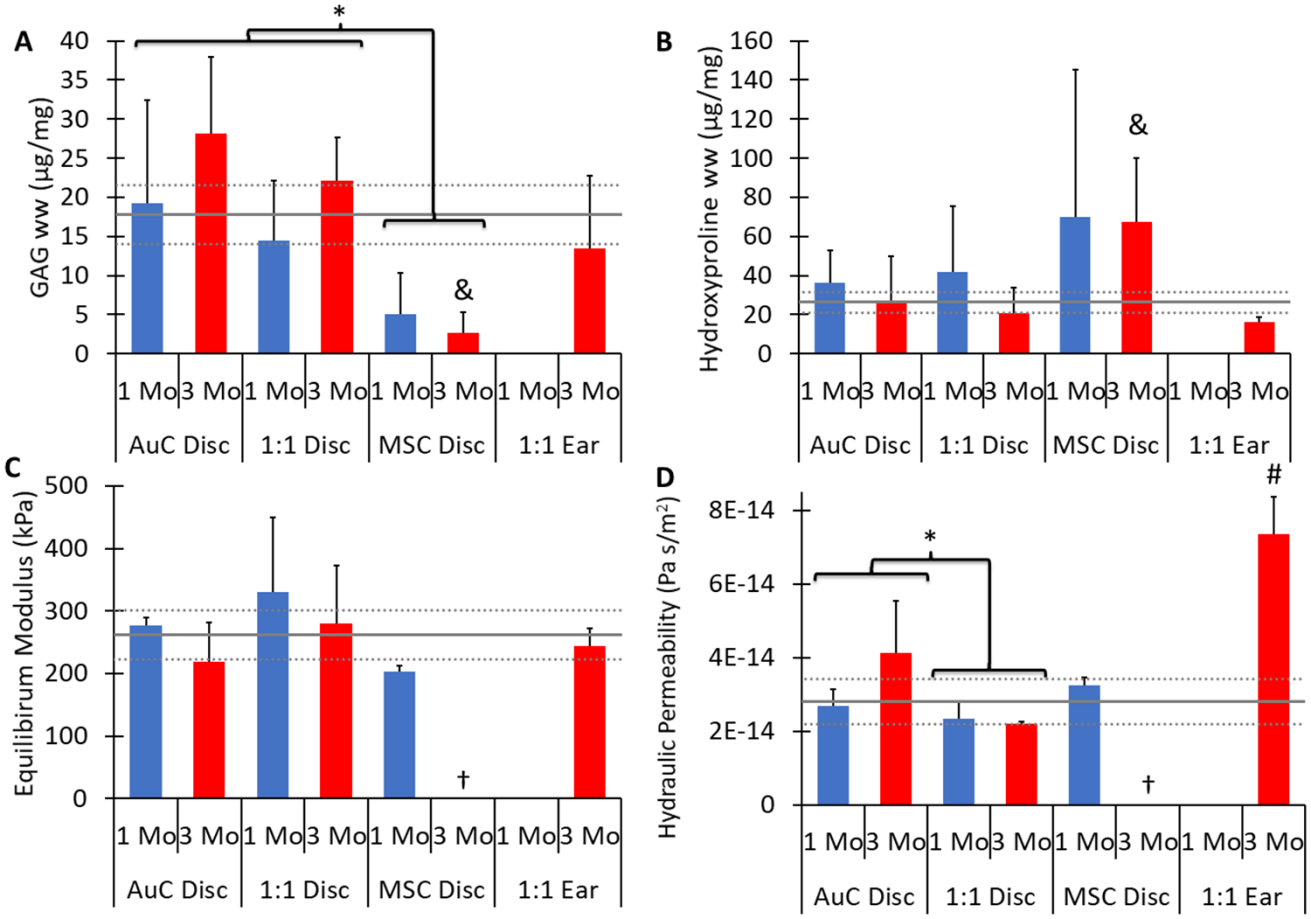
Cartilage composition and biomechanics. (**A**) Glycosaminoglycan (GAG) deposition was significantly higher for AuC and 1:1 discs compared to MSC discs. The amount of GAG in AuC and 1:1 discs at 3 months and 1:1 ear constructs was similar to native human auricular cartilage. (**B**) Hydroxyproline content, representing both collagen and elastin, was significantly lower for AuC and 1:1 discs compared to MSC discs, and similar for AuC and 1:1 discs at 3 months with 1:1 ear constructs and native cartilage. For both (A) and (B), n = 6–9 and all data are normalized to tissue wet weight (ww). (**C**) Equilibrium modulus was similar for all disc and ear constructs compared to native human auricular cartilage. (**D**) The hydraulic permeability was similar for all disc constructs compared to native cartilage, but was significantly higher for 1:1 ear constructs compared to all other samples. For both (C) and (D), n = 2–4, † indicates that 3 month MSC samples were too small to undergo testing. For all data, solid gray line indicates native human auricular cartilage, dashed gray line indicates ± one standard deviation, * indicates significant difference in cell type, & indicates significant difference from AuC disc, 1:1 disc, and 1:1 ear at 3 months, # indicates significant difference from AuC disc (3 month), 1:1 disc (3 month), and native tissue, *P* < 0.05. Data displayed as mean + one SD. No 1 month 1:1 ear constructs were included in this study.

Monocellular AuC discs and combined cellular 1:1 discs and ears displayed GAG content similar to native human ear cartilage following 3 months *in vivo*. AuC discs and 1:1 discs generated significantly more GAGs than MSC discs (Fig 4A, *P* < 0.001). The GAG content of AuC and 1:1 discs displayed an increasing trend with time, although no statistical difference was observed between 1 and 3 months (*P* = 0.13). At 3 months, AuC discs contained 158% the GAG content of native auricular cartilage, and 1:1 discs contained 125% native GAG content. MSC discs, however, contained only 15% the GAG content of native cartilage, indicating almost no chondrogenic potential of the stem cells in isolation. Full-scale 1:1 ear constructs displayed GAG content at 3 months similar to AuC and 1:1 discs, containing 76% that of native auricular cartilage, which was not significantly different (Fig 4A, *P* = 0.998).

After 3 months of implantation, the hydroxyproline contents of AuC discs, 1:1 discs, and 1:1 ears were similar to native auricular cartilage. AuC discs at 3 months had 104% of the hydroxyproline content of native cartilage, while 1:1 discs at 3 months had 79% of the native hydroxyproline content (Fig 4B). By contrast, MSC discs, which failed to generate elastin or proteoglycans, contained 257% of the native hydroxyproline at 3 months, significantly higher than other disc groups (*P* > 0.05). The higher concentration of hydroxyproline corresponds to the dense collagen fibers observed in the MSC disc tissues in histology (Fig 3J). No differences with time were observed (*P* = 0.23). Ear constructs containing the 1:1 cell ratio did not display significantly different hydroxyproline contents from AuC or 1:1 discs (*P* = 0.83, *P* = 0.99, respectively). Ear constructs featured 61% of the hydroxyproline content of native auricular cartilage, which was not significantly different, and may be a result of the less developed elastin network present in these tissues (Fig 4B, *P* = 0.94).

The equilibrium modulus of all disc and ear constructs was similar to that of native cartilage. The equilibrium modulus was not significantly different between the AuC, 1:1, and MSC discs (*P* = 0.14), and no differences were observed between time points (Fig 4C, *P* = 0.41). The MSC discs at 1 month had lower equilibrium moduli than the other disc constructs or native cartilage, but this difference was not significant. Three months following implantation, MSC discs had contracted to diameters less than the 3 mm minimum for the confined compression chamber used, and therefore could not be mechanically evaluated. Full-scale 1:1 ear constructs featured equilibrium moduli that were not significantly different from AuC or 1:1 disc constructs or native cartilage (Fig 4C, *P* < 0.74). In addition to confined compression, the gross flexibility of the full ear constructs was investigated through bending. Bending of the constructs by hand following explanation demonstrated the elastic properties of the engineered 1:1 ears (S1 Movie).

AuC, 1:1, and MSC discs displayed similar hydraulic permeability to that of native auricular cartilage, while 1:1 ears were significantly greater. Hydraulic permeability is a measure of the ease with which water moves through the tissue, and an indicator of the density of the proteoglycan network. The hydraulic permeability of the disc constructs showed no significant differences between time points (Fig 4D, *P* = 0.20), but was significantly different between AuC and 1:1 discs (*P* = 0.02). All disc constructs were within 26% the permeability of native cartilage. Full-scale ear constructs displayed a significantly higher hydraulic permeability than discs or native ear cartilage, featuring a permeability 261% higher than native tissue after 3 months *in vivo* (Fig 4D, *P* < 0.05).

## Discussion

The objective of this study was to generate a full-scale human auricular cartilage implant using cells derived from a clinically accessible amount of donor ear cartilage by utilizing human MSCs to supplement human AuCs. The data from this study show that engineered constructs fabricated using a 1:1 ratio of human AuCs and MSCs generated cartilage *in vivo* that was equivalent to native auricular cartilage and constructs containing solely AuCs, whereas constructs with only MSCs failed to generate cartilage tissue. Based on these promising results, we generated full-scale ear constructs using exclusively human cells in a 1:1 AuC:MSC ratio, which generated native-like auricular cartilage following 3 months of subcutaneous implantation.

Currently, one of the main obstacles to translating large-scale tissue and organ engineering to the clinic is obtaining a sufficient cell source of autologous cellular components. Our previous work successfully demonstrated the potential of combining CAD/CAM technology and injection molding using high density collagen to produce patient-specific tissue engineered auricles [5,11]. However, the translational impact of those studies was limited by the use of neonatal bovine cartilage as the cell source. The optimal clinical cell source is autologous AuCs, isolated from either the microtic cartilage remnant [9,21,33] or a non-deforming biopsy of the contralateral ear, yielding approximately 1 g of elastic cartilage [34], similar to those used in this study. Samples this size provided ~10 million cells, similar to previous findings [35], but still insufficient to populate a pediatric-sized ear requiring >200 million cells [5,11]. Human AuCs proliferate *in vitro*, but like other chondrocytes, can dedifferentiate when cultured in monolayer [2]. Expansion through third passage, as performed here, provided ~115 million AuCs, only half of the requirement to produce a human ear. As such, there exists a need for an alternative, clinically accessible cell source to replace or supplement AuCs in auricular cartilage engineering.

The burden of acquiring large numbers of AuCs can be reduced by combining them with MSCs, which are readily available from bone marrow and expand to great numbers in culture [14,15]. Almost 40 studies have utilized MSCs for the clinical repair of cartilage, primarily articular cartilage [36]. Co-culture of MSCs with articular chondrocytes [19], meniscal fibrochondrocytes [17,18], and nucleus pulposus cells [20] all resulted in enhancement of cartilage development. Co-culture and co-implantation of MSCs with AuCs has also been demonstrated with animal cells [23,25] and human-animal cell hybrids [21,22]. Only one previous co-implantation study combined both AuCs and stem cells of human origin, co-implanting AuCs with adipose-derived stem cells [24]. Similar to previous co-implantation studies, we observed comparable auricular cartilage generation between monocellular AuC and combined cellular implanted discs, while we also demonstrated that co-implantation of human cells can be extended to a full-scale ear construct.

Importantly, this study demonstrated that auricular cartilage generated in the 1:1 discs was similar in structure, biochemical development, and mechanical properties to both discs containing 100% AuCs and native human auricular cartilage after only 3 months *in vivo*. Based on these studies, we fabricated full-scale ear constructs containing exclusively human cells using the 1:1 co-implant ratio. Histologic analyses of the 1:1 ear constructs demonstrated the generation of auricular cartilage *in vivo*, including the development of key structures such as the perichondrial layer, a proteoglycan rich interstitium, and formation of cellular lacunae. Although the elastin network was not as well developed as those of the disc constructs at the 3 month time point, the appearance of early elastin fibers is a critical indicator of the auricular cartilage phenotype. The level of elastin development is also in agreement with the findings of other studies that examined full-scale engineered ear constructs with non-100% human cell sources at similar time points [7,23]. Most importantly, the 1:1 ear constructs featured similar biochemical and mechanical properties to native auricular cartilage.

In comparison to other auricular cartilage co-implantation studies, our work improved cellular efficiency and demonstrated potential for long-term stability of the engineered cartilage. Previous research has focused on increasing the ratio of MSCs used to supplement AuCs, with ratios up to 3:7 AuCs to MSCs generating cartilage [24]. However, these constructs also required an initial cell concentration of 50 million cells/mL, twice the density of the constructs in the present study. The full-scale ear constructs presented here required only 12.5 million AuCs/mL, less than the 25 million AuCs/mL needed for a monocellular implants [11] or the 15 million AuCs/mL [24] achieved in other co-implant work. Over the course of life, auricular cartilage does not undergo the same mechanical loading as hyaline or fibrocartilage, but the auricle still endures deformation in the form of bending, compression, and tension. Engineered constructs are also exposed to tension of the skin during implantation and development. Unlike previous co-implant studies [22,24], this study further characterized the compressive properties of the engineered cartilage, finding the tissue to be mechanically similar to native ear cartilage, and subjected the ear constructs to bending, with the ears elastically returning to their initial conformation. These data demonstrate that the engineered constructs remain mechanically stable under mechanical stress during the development period *in vivo*, and that they feature sufficient stiffness to endure mechanical exposure following reconstruction.

To the best of our knowledge, only one previous study has combined AuCs and MSCs to form an auricle [21], and no group has previously reported the use of both chondrocytes and stem cells from human sources to generate human ear-shaped auricular cartilage. These data demonstrate the clinical viability of human cell sources and the co-implant framework to fabricate a tissue engineered auricle. We isolated human AuCs from ear cartilage samples of less than 1 gram, taken from a diverse population of donors, all of which were expanded through three passages without losing the capacity to generate auricular cartilage *in vivo*. In contrast, previous studies found that expanded human AuCs failed to generate cartilage *in vivo* when cultured with primary cells or in medium conditioned by primary cells [37], and failed to develop elastin when cultured with or without serum [34]. Pellet cultures of microtia-derived AuCs at P3 also displayed a decline in chondrogenic phenotype during *in vitro* culture [21]. Disc constructs containing solely human AuCs at P3 in this study formed tissue similar to native auricular cartilage, without the need of growth factor treatments during expansion or *in vitro* culture. However, discs containing solely human MSCs failed to generate auricular cartilage during subcutaneous implantation, further reinforcing the findings that neocartilage formation and chondrogenic differentiation of the MSCs in the co-implant constructs is directed by the inclusion of AuCs [23,24].

The process of tissue engineering a human auricle described here closely parallels current clinical techniques for repairing focal defects of articular cartilage, which has been in clinical practice for two decades. Initial autologous chondrocyte implantation (ACI) procedures, developed to treat large defects, isolated and cultured autologous articular chondrocytes before implanting them within the site of injury in the patient [10]. This process was further enhanced by seeding or embedding the autologous cells within a membrane or scaffold, such as collagen, prior to implantation, and is now in clinical trials within the United States and available clinically in Europe [10,38,39]. Like ACI, we isolated chondrocytes from healthy cartilage, then expanded these chondrocytes before encapsulating them in a collagen matrix and implanting the full construct. However, the process of forming a full-scale ear implant requires significantly greater tissue generation compared to ACI. The pediatric 1:1 auricles generated ~5 mL of cartilage after 3 months *in vivo*, a much greater amount than the 0.5–2 mL of tissue needed to fill large defects of the articular surface [40,41]. One other study has generated a similarly sized auricle using a clinical biopsy of ear cartilage as an initial cell source, generating tissue with auricular cartilage structure following implantation in 5 pediatric human patients [9]. The construct generation process required a 12 week period of *in vitro* culture prior to implantation for the seeded cells to attach, infiltrate, and expand within the synthetic polymer scaffold, significantly delaying the time to patient delivery. In contrast, by using a high-density collagen hydrogel with cells encapsulated homogeneously, we can go directly from construct generation to implantation without the need for extensive time in culture. Additionally, the clinical application of the co-implantation method is supported by the increasing use of MSCs in clinical trials of articular cartilage repair, demonstrating the accessibility and safety of MSCs for cartilage engineering and regeneration [42].

While the successful generation of auricular cartilage in this study is encouraging, several limitations must still be addressed. The auricle is an external organ which funnels sound into the ear canal, and deformation can result in conductive hearing loss [43]. In addition, the abnormal appearance associated with auricular deformation can cause significant psychological distress in pediatric patients [43]. All disc and ear constructs in this study displayed significant contraction from the initial dimensions during subcutaneous implantation, with the discs displaying a greater reduction in size compared to similar constructs containing cells of bovine origin [23]. The ear constructs shrank to nearly half of the pre-implantation size, and this contraction may have also contributed to the loss of some patient-specific morphological details. Contraction is commonly observed in cell seeded collagen constructs [5,27,44], and can be addressed in several manners. Potential solutions include enlarging the initial constructs to account for contraction or the addition of a cross-linking agent such as riboflavin [44,45], which has been demonstrated to reduce contraction without affecting cell viability. Additionally, *in vitro* culture of the constructs prior to implantation could allow for early maturation of the construct without exposure to compression or tension from the skin. Culture under hypoxic conditions has been shown to increase proteoglycan and type II collagen synthesis and increase lysyl oxidase crosslinking, which could enhance initial mechanical properties and prevent further contraction from occurring [12,46–48].

Additionally, implantation for longer time points and increased sample number could improve the significance of this study. The elastin network for the engineered discs was not as dense at 3 months as that of native tissue. However, the fibers stained much more strongly than for discs after only 1 month *in vivo*, while the perichondrial layer and proteoglycans were already apparent at this time. This corresponds with the slow turnover time of elastin relative to other matrix molecules [49], and elastin fibers may develop further with increased time *in vivo*. The 1:1 ear constructs displayed heterogeneous deposition of proteoglycans and elastin with a lower GAG content relative to native auricular cartilage tissue, which may be a result of the increased tissue thickness limiting diffusion and nutrient access. However, the presence of healthy cells within the tissue and the development trend observed from 1 to 3 months in the disc constructs indicates that these tissues could generate more proteoglycan-rich tissue with mature elastin fibers over time. We have previously observed similar results in longer-term implantation studies of ear constructs containing bovine cells, where by 6 months the ears featured auricular cartilage microstructure throughout the tissue [5,6]. Implantations for longer time points are also critical to demonstrate the stability of these tissue engineered ears. Finally, other co-implantation studies have investigated up to 1:4 AuCs to MSCs to generate auricular cartilage [21,22,24], although a ratio of 1:9 AuCs to MSCs failed to produce cartilaginous tissue [25]. The success of the 1:1 constructs in this study may extend to lower ratios, allowing for more significant supplementation with the clinically accessible MSCs.

Beginning with a small, clinically relevant sample of patient ear cartilage, a large number of AuCs were isolated and expanded without the loss of chondrogenic phenotype. These were then combined with MSCs, which are obtainable with minimally invasive surgery from the patient. By combining these cell sources, we recreated a human-shaped, patient-specific ear, that generated human auricular cartilage following implantation. The results of this study demonstrate the feasibility of tissue engineering human auricles as a superior clinical option for auricular reconstruction.

## Materials and methods

### Experimental design

This study was designed to investigate the hypothesis that human mesenchymal stem cells (MSCs) could be combined with human auricular chondrocytes (AuCs) to generate auricular cartilage tissue *in vivo*. Full-sized, pediatric ear constructs were generated with a combination of AuCs and MSCs to demonstrate tissue generation on a clinically relevant scale and retention of the human ear aesthetic following maturation. Cylindrical disc constructs were generated and implanted as surrogates for full-sized ear constructs to compare tissue generation of cell combinations to monocellular constructs featuring either human AuCs or human MSCs. Tissue generation was determined by gross morphology and structural, compositional, and mechanical development. Sample size for the disc constructs were based on previous studies [23], and sample size for the ear constructs were based on availability of human cartilage tissue. Data collection following 1 and 3 month implantation time points was predetermined. Constructs complicated by wound infection or seroma formation were excluded from analysis. No outliers were excluded from analyses, but biochemical data were square root transformed and hydraulic permeability data were log transformed prior to statistical analyses. For disc constructs, each sample was tested once for biochemical and mechanical data. Ear constructs were sampled and tested in at least triplicate for biochemical and mechanical data. Human ear tissue was acquired from 3 separate patients for disc constructs and 5 separate patients for ear constructs, with a single source line of human MSCs used for all constructs. Statistical analyses took into account host animal as a random variable. Investigators were not blinded in this study.

### Ethics statement

All animal care and experimental procedures were in compliance with the Guide for the Care and Use of Laboratory Animals [50] and were approved by the Weill Cornell Medical College Institutional Animal Care and Use Committee (protocol # 2011–0036). Animals were stored in an approved xenograft room for immunodeficient animals and kept in clear plexiglass/vented boxes with pellet food and water provided ad libitum. Animals were housed together prior to surgery (3–4 per cage) then singly following implantations. The facility was kept at 21°C with a 12 hour light/dark cycle. All animals were given environmental enrichment with paper bedding and nylabones. Human ear cartilage remnants were obtained by Drs. Charles Thorne and John Sherman at their private practices. Tissue samples used were not collected for the purpose of the study and were considered clinical waste. Based on the consultation with Human Research Protection Program and Division of Research Integrity at Weill Cornell Medicine, it was determined that the use of this tissue was not considered human subjects research and did not require IRB review.

### Isolation and expansion of human auricular chondrocytes

Human auricular chondrocytes were isolated based on methods previously described [11]. Briefly, ear cartilage remnants from otoplasty procedures were obtained in New York City and received in Ithaca, NY on the same day as surgical excision (S1 Table). Auricular cartilage was dissected from the perichondrium under sterile conditions. Samples of cartilage from a subset of patients were fixed for histological staining or frozen for biochemical and mechanical analyses. Cartilage for chondrocyte extraction was diced into 1 mm^3^ pieces and digested overnight in 0.2% collagenase (Worthington Biochemicals Corp., Lakewood, NJ), 100 μg/ml penicillin, and 100 μg/ml streptomycin in Dulbecco’s modified Eagle’s medium (DMEM) (MediaTech Inc., Manassas, VA).

AuCs were filtered, washed with 1x phosphate buffered saline (PBS) (MediaTech) with 100 μg/ml penicillin and 100 μg/ml streptomycin, and counted the following day. AuCs were plated at approximately 10,000 cells/cm^2^ and cultured in DMEM containing 10% fetal bovine serum (FBS) (Gemini Bio-Products, Sacramento, CA), 100 μg/ml penicillin, 100 μg/ml streptomycin, and 0.1 mM non-essential amino acids under 5% pCO_2_ and 37 °C. AuCs were expanded through third passage (P3), using 0.25% trypsin (MediaTech) to release cells between passages.

### Expansion of human mesenchymal stem cells

A human mesenchymal stem cell line (Lot 0017, RoosterBio Inc., Frederick, MD) was expanded under 5% pCO_2_ and 37 °C to population doubling level 14–15. Cells were expanded in hBM-MSC High Performance Media (RoosterBio Inc.). MSCs were released with 0.25% trypsin and washed with PBS with 100 μg/ml penicillin and 100 μg/ml streptomycin between passages.

### Hydrogel disc construct fabrication

Collagen was extracted and reconstituted as previously described [51,52]. At the time of fabrication, stock collagen solution was returned to pH 7.0 and maintained at 300 mOsm by mixing with appropriate volumes of 1N NaOH (Sigma-Aldrich, St. Louis, MO), 10x PBS, and 1x PBS as previously described [26]. Collagen solution was immediately mixed with cells suspended in PBS and formed into disc constructs as previously described [17,23]. Briefly, cell suspensions were formed with AuC:MSC ratios of 1:0, 1:1, and 0:1. Neutralized collagen was then homogeneously mixed with the cell suspensions for a final cell concentration of 25 x 10^6^ cells/mL and collagen density of 10 mg/mL. The collagen hydrogel was extruded between two glass plates spaced 2 mm apart and allowed to undergo thermal gelation at 37 °C for 1 hour. Following gelation, 8 mm diameter disc constructs were formed using a dermal biopsy punch and placed in the same media used for cell expansion. Constructs were implanted within 48 hours.

### Pediatric ear construct fabrication

A pediatric ear mold and collagen hydrogel ear constructs were formed as previously described [11]. Briefly, neutralized collagen solution was immediately mixed with a cell suspension containing AuCs and MSCs in a 1:1 ratio. The collagen hydrogel with a final cell concentration of 25 x 10^6^ cells/mL and collagen density of 10 mg/mL was injected into ear molds and allowed to undergo thermal gelation at 37 °C for 1 hour. Following gelation, ear constructs were removed from the molds and cultured for a maximum of 1 week in media composed of DMEM, 10% FBS, 100 μg/ml penicillin, 100 μg/ml streptomycin, and 0.1 mM non-essential amino acids prior to implantation.

### Construct implantation and explantation

Disc constructs were implanted as previously described [23]. Briefly, 10-week old male athymic nude mice (NU/NU; Charles River, Wilmington, MA) weighing between 20–25 g were anesthetized and prepped. Three 1 cm incisions were made on the dorsum of each mouse to create a subcutaneous pocket and one of the 1:0, 1:1, and 0:1 AuC:MSC ratio discs was implanted in each pocket per mouse. Animals were sacrificed 1 or 3 months after implantation. Discs were harvested, weighed, imaged, and measured for final height and diameter. A representative set of specimens were fixed in 10% neutral buffered formalin for 48 hours and transferred to 70% ethanol prior to histologic analyses. The remainder were snap frozen in liquid nitrogen for biochemical and biomechanical analyses. A total of 77 discs (24 1:0, 27 1:1, and 26 0:1 AuC:MSC) were recovered. Implants complicated by seroma formation were excluded from analyses.

Ear constructs were implanted as previously described [5,11]. Briefly, 10-week old male athymic nude rats (RNU; Charles River, Wilmington, MA) weighing between 300–400 g were anesthetized and prepped. A subcutaneous pocket overlying the dorsum was dissected and a single 1:1 AuC:MSC cell ratio ear construct was implanted. Animals were sacrificed 3 months after implantation. Constructs were harvested, weighed, imaged, measured, and halved. Construct measurements were performed as previously described [11]. Briefly, length was measured along the lobule-helix axis, and width was defined as the largest distance measured perpendicular to the lobule-helix axis. Half of each specimen was fixed in 10% neutral buffered formalin for 48 hours and transferred to 70% ethanol prior to histologic analyses. The remainder was snap frozen in liquid nitrogen for biochemical and biomechanical analyses. A total of 6 ear constructs were recovered. Implants complicated by seroma formation were excluded from analyses.

A total of 27 mice and 8 rats were used in this study, all drug and test naïve. There were no indications of animals having trouble recovering from the implantations, nor did any animals display signs of illness. Animals were monitored daily for the first 3 days post-implantation, then three time per week until sacrifice. No animals died without euthanasia as a result of experimental procedures. Both mice and rats were sacrificed by CO_2_ asphyxiation. Rats also received a pneumothorax creation. Anesthesia was administered intraperitoneally and consisted of ketamine and xylazine cocktail dosed by weight, and analgesia by buprenorphine and meloxicam dosed by weight. Ketamine/xylazine cocktail was chosen to provide ample time for surgical procedures with limited side effects and administered for fast onset. Dosing for mice was 80–100 mg/kg IP (ketamine) and 10–12.5 mg/kg IP (xylazine. Dosing for rats was 40–100 mg/kg IP (ketamine) and 5–13 mg/kg IP (xylazine). During surgery, animals were observed for visible signs of distress, including increased respiration, blink reflex, or reaction to toe and tail pinch. If the level of anesthesia was found to be inadequate, a booster dose of half the original amount was given and the animal was reassessed. Animal vitals and condition were recorded in an anesthesia log every 15 minutes. Surgical prep included shaving and prep with betadine and alcohol. The duration of surgeries was approximately 30 minutes. Surgeries were performed in the afternoon in a biosafety cabinet in a xenograft room controlled for animals with increased sterility needs. Sutures were removed 11 days post-operation.

### Histological analyses

Fixed samples were dehydrated by sequential washes in ethanol, embedded in paraffin, and cut into 5 μm sections. Sections were stained with Safranin O/Fast green to assess proteoglycan distribution, Picrosirius red to assess collagen organization, and Verhoeff’s/Van Gieson to assess the presence of elastin fibers. Images were taken in brightfield at 100x, 200x, and 400x using a Nikon Eclipse TE2000-S microscope (Nikon Instruments, Melville, NY) fitted with a SPOT RT camera (Diagnostic Instruments, Sterling Heights, MI). Scans of 1:1 full ear constructs were also taken using an Aperio ScanScope CS2 at 20x magnification (Leica Biosystems Inc, Buffalo Grove, IL).

### Biochemical analyses

Biochemical analyses were performed as previously described [53]. Briefly, full discs were collected for the disc constructs or three samples collected from each ear for the full-scale ear constructs. Samples from human ear cartilage remnants were also collected and analyzed. Samples were weighed, frozen, lyophilized, and weighed again. Samples were then digested with 1.25 mg/ml papain (Sigma-Aldrich) solution overnight at 60°C and analyzed for DNA content via the Hoechst DNA assay [28], sulfated glycosaminoglycan (GAG) content via a modified 1,9-dimethylmethylene blue (DMMB) assay [29], and collagen and elastin via a hydroxyproline assay [30,31]. Biochemical properties are reported normalized to sample wet weight (WW).

### Mechanical analysis

Three mm diameter by one mm height cylinders were cut from the central portion of each disc construct, or three samples were taken from each ear construct using dermal biopsy punches. Cylinders were also taken from human ear cartilage remnants. Confined compression testing was performed as previously described [23,32]. Briefly, samples were thawed in PBS containing protease inhibitors (Roche Diagnostics, Indianapolis, IN) and placed in a cylindrical confining chamber mounted in an ELF 3200 test frame (Enduratec, Eden Prarie, MN). Samples were compressed to 50% of their original height in 10 steps of 50 μm each, with 5 minutes between steps to allow for full stress relaxation. Resultant stresses were recorded at 1 Hz and the temporal profiles of stress were fit to a poroelastic model of tissue behavior using custom MATLAB (MathWorks, Natick, MA) code to calculate the equilibrium modulus and hydraulic permeability [53].

### Statistical analysis

All statistical analysis was performed using RStudio (RStudio, Boston, MA). AuC expansion data was analyzed by a one-way ANOVA on ranks with Dunn’s Method pairwise multiple comparison. Engineered disc constructs were analyzed using a mixed effect model with random (mouse) and fixed (time, cell group) effects with a Tukey post hoc test. Fixed effect interaction term was removed when non-significant to simplify model. Disc constructs at 3 months were also compared to full ear constructs at 3 months and native human auricular cartilage by one-way ANOVA with Tukey post hoc test. All data are represented as mean plus one standard deviation. *P* values < 0.05 are considered statistically significant.

## Supporting information

S1 Fig Engineered auricular cartilage microstructure after 1 month. Histological staining of engineered disc constructs containing AuC:MSC ratios of 1:0 (**A-C**), 1:1 (**D-L**), and 0:1 (**M-O**) following 1 months *in vivo*. Picrosirius Red staining (**A, D, G**) displayed the formation of a perichondrium (PC) composed of collagen fibers on the perimeter of AuC and 1:1 discs, while MSC discs were composed of fibrous collagen throughout. Safranin O staining with Fast Green counterstain (**B, E, H**) displayed proteoglycan deposition and cell lacunae formation in AuC and 1:1 discs, with no proteoglycan deposition in MSC discs. Verhoeff’s stain (**C, F, I**) displayed limited formation of elastic fibers (EF) in AuC discs, while 1:1 and MSC discs did not display elastin fibers after 1 month. Scale bar = 100 μm.
(TIF)

S2 Fig Engineered cartilage water and cellular content. (**A**) The DNA content, representing cellular content of the tissue, was not significantly different between disc constructs, nor between discs and full ear constructs from native human ear. DNA content was normalized to tissue wet weight (ww). (**B**) Water content was significantly higher for AuC and 1:1 discs compared to MSC discs. At 3 months, the hydration of MSC discs was significantly less than other disc constructs and full ear constructs, but constructs were not significantly different from native ear cartilage. For all data, n = 6–9, solid gray line indicates native human auricular cartilage, dashed gray line indicates ± one standard deviation, * indicates significant difference in cell type, & indicates significant difference from AuC disc, 1:1 disc, and 1:1 ear at 3 months, *P* < 0.05. Data are displayed as mean + one SD. No 1 month 1:1 ear constructs were included in this study.
(TIF)

S1 Table Demographics of the patient donor population. All cells from a single patient were used in either one set of disc constructs or one ear construct.
(DOCX)

S1 Movie Demonstration of full-scale ear bending. Elastic bending response of 1:1 ear construct following 3 month implantation.
(M4V)

S1 File Human AuC Expansion log.xlsx. Human auricular chondrocyte expansion dataset.
(XLSX)

S2 File Biochem Data Collected.xlsx. Biochemical analyses dataset.
(XLSX)

S3 File Biomechanics Data Collected.xlsx. Biomehcanical analysis dataset.
(XLSX)

S4 File Disc Construct Size Data.xlsx. Disc construct gross size analysis dataset.
(XLSX)

S5 File Ear Construct Size Data.xlsx. Ear construct gross size analysis dataset.
(XLSX)

S6 File Raw Mechanical Data.zip. Raw data outputs for confined compression testing.
(ZIP)

S7 File NC3Rs ARRIVE Guidelines Checklist Cohen.pdf. Completed checklist of animal care.
(PDF)
