# Supplementary figures and images for: Tissue engineering the human auricle by auricular chondrocyte-mesenchymal stem cell co-implantation
Source: PLoS One. 2018 Oct 24;13(10):e0202356. doi: 10.1371/journal.pone.0202356 (PMC6200177; doi:10.1371/journal.pone.0202356)

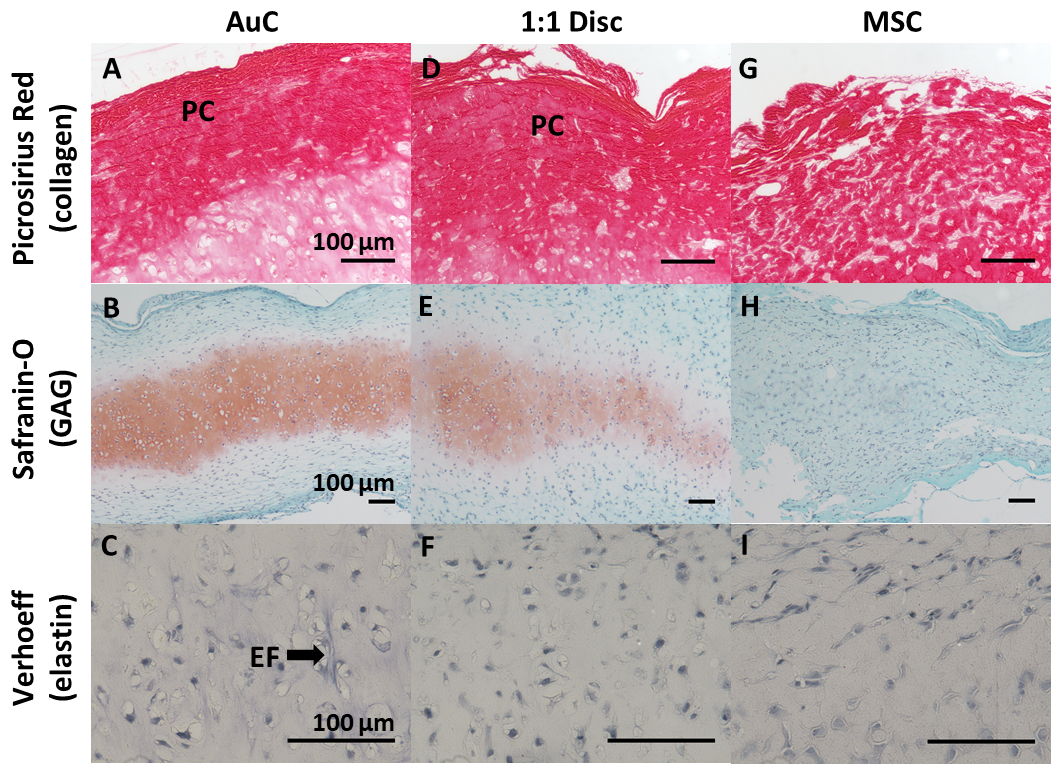

Supplement: S1 Fig — Histological staining of engineered disc constructs containing AuC:MSC ratios of 1:0 (A-C), 1:1 (D-L), and 0:1 (M-O) following 1 months in vivo. Picrosirius Red staining (A, D, G) displayed the formation of a perichondrium (PC) composed of collagen fibers on the perimeter of AuC and 1:1 discs, while MSC discs were composed of fibrous collagen throughout. Safranin O staining with Fast Green counterstain (B, E, H) displayed proteoglycan deposition and cell lacunae formation in AuC and 1:1 discs, with no proteoglycan deposition in MSC discs. Verhoeff’s stain (C, F, I) displayed limited formation of elastic fibers (EF) in AuC discs, while 1:1 and MSC discs did not display elastin fibers after 1 month. Scale bar = 100 μm. (TIF) [file pone.0202356.s001.tif]

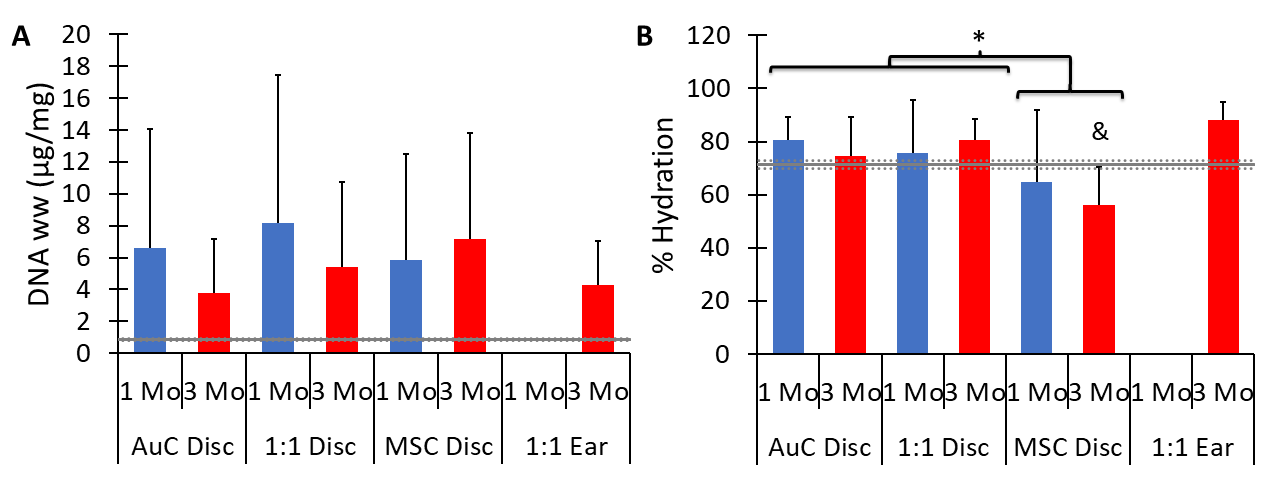

Supplement: S2 Fig — (A) The DNA content, representing cellular content of the tissue, was not significantly different between disc constructs, nor between discs and full ear constructs from native human ear. DNA content was normalized to tissue wet weight (ww). (B) Water content was significantly higher for AuC and 1:1 discs compared to MSC discs. At 3 months, the hydration of MSC discs was significantly less than other disc constructs and full ear constructs, but constructs were not significantly different from native ear cartilage. For all data, n = 6–9, solid gray line indicates native human auricular cartilage, dashed gray line indicates ± one standard deviation, * indicates significant difference in cell type, & indicates significant difference from AuC disc, 1:1 disc, and 1:1 ear at 3 months, P < 0.05. Data are displayed as mean + one SD. No 1 month 1:1 ear constructs were included in this study. (TIF) [file pone.0202356.s002.tif]
