# Supplementary material for: Tissue engineering the human auricle by auricular chondrocyte-mesenchymal stem cell co-implantation
Source: PLoS One. 2018 Oct 24;13(10):e0202356. doi: 10.1371/journal.pone.0202356 (PMC6200177; doi:10.1371/journal.pone.0202356)
Supplement: S1 Table — All cells from a single patient were used in either one set of disc constructs or one ear construct. (DOCX) [file pone.0202356.s003.docx]

| **Gender** | **Age** | **Construct Type** |
| --- | --- | --- |
| Male | 10 | Disc |
| Male | 14 | Disc |
| Female | 38 | Disc |
| Male | 29 | Ear |
| Female | 33 | Ear |
| Female | 39 | Ear |
| Female | 42 | Ear |
| Female | 49 | Ear |
